# Learning from Demonstrations via Deformable Residual Multi-Attention Domain-Adaptive Meta-Learning

**DOI:** 10.3390/biomimetics10020103

**Published:** 2025-02-11

**Authors:** Zeyu Yan, Zhongxue Gan, Gaoxiong Lu, Junxiu Liu, Wei Li

**Affiliations:** 1 The Academy for Engineering and Technology, Fudan University, Shanghai 200433, China; zeyuyan22@m.fudan.edu.cn (Z.Y.); gxlu22@m.fudan.edu.cn (G.L.); 2 School of Electronic Engineering, Guangxi Normal University, Guilin 541001, China; liujunxiu@gxnu.edu.cn

**Keywords:** meta-learning, learning from demonstrations, one-shot learning, deep learning in grasping and manipulation

## Abstract

In recent years, the fields of one-shot and few-shot object detection and classification have garnered significant attention. However, the rapid adaptation of robots to previously unencountered or novel environments remains a formidable challenge. Inspired by biological learning processes, meta-learning seeks to replicate the way humans and animals quickly adapt to new tasks by leveraging prior knowledge and generalizing across experiences. Despite this, traditional meta-learning methods that rely on deepening or widening neural networks offer only marginal improvements in model performance. To address this, we proposed a novel framework termed Residual Multi-Attention Domain-Adaptive Meta-Learning (DRMA-DAML). Our framework, motivated by biological principles like the human visual system’s concurrent handling of global and local details for enhanced perception and decision making, empowers the model to significantly enhance performance without augmenting the depth of the neural network, thus avoiding the overfitting and vanishing gradient problems typical of deeper architectures. Empirical evidence from both simulated environments and real-world applications demonstrates that DRMA-DAML achieves state-of-the-art performance. Specifically, it improves adaptation accuracy by 11.18% on benchmark tasks and achieves a 97.64% success rate in real-world object manipulation, surpassing existing methods. These results validate the effectiveness of our approach in rapid adaptation for robotic systems.

## 1. Introduction

In the quest for swift adaptation to diverse tasks, settings, or environments, meta-learning [1] has been extensively explored within the realms of one-shot or few-shot target detection [2], classification [3], speech recognition [4], and image segmentation [5]. Despite these advancements, the challenge of training a robot to reliably adapt to previously unencountered scenarios or novel environments through learning from demonstration (LfD) persists. This endeavor is fraught with complexities, as traditional meta-learning approaches that hinge on deepening or broadening neural networks yield only marginal improvements in model efficacy.

Drawing upon the principles of imitation learning [6,7] and the sophisticated processing capabilities of Long Short-Term Memory (LSTM) networks [8], a meta-learning framework [9], combined with the soft-attention mechanism, is proposed for stacking tasks. This framework is inspired by the model-agnostic meta-learning (MAML) approach [10], and a meta-imitation learning (MIL) framework proposed in [11] has demonstrated efficacy in simulated reaching, simulated pushing, and real-world placing tasks, significantly surpassing the performance benchmarks set in [9]. In comparison to previous MAML-based endeavors [12,13], introduced Task-Embedded Control Networks (TecNets), which are grounded in metric learning [12,14]. To facilitate robots in executing tasks through the visual observation of human demonstrations, a Domain-Adaptive Meta-Learning (DAML) framework, which is based on MIL and MAML, was proposed in [15]. By incorporating a target recognition module into DAML, the Target Recognition-Based Meta-Imitation Learning (TaR-MIL) approach presented in [16] is utilized to discern the target object amidst other distractors. Acknowledging the human capacity to learn across diverse domains, Random Domain-Adaptive Meta-Learning (RDAML) [17] explores the robot’s ability to learn from both human and robotic demonstrations. Similarly to TecNets, Replayed Task Contrastive Model-Agnostic Meta-Learning (RTMAML) [18] was proposed to instruct the robot in distinguishing tasks involving positive and negative samples or demonstrations. This advanced framework represents a significant stride in the field of meta-learning, offering a robust and adaptable approach to task learning and execution.

Acknowledging that the conventional approach of enhancing model complexity through the deepening or widening of neural networks, as per previous meta-learning methodologies, yields only marginal improvements in performance, a novel framework was introduced: Multi-Level and Multi-Attention Domain-Adaptive Meta-Learning (MLMA-DAML) [19].

This framework underscores the significance of shallow features extracted by neural networks in bolstering model performance. The marginal gains in model performance when deepening or widening neural networks may stem from the gradual neglect or loss of shallow features as the network complexity increases—features that are pivotal in the meta-learning adaptation process. Additionally, the model’s generalization capability on the test set may be compromised due to an overfitting tendency to the training set.

**Figure 1:**
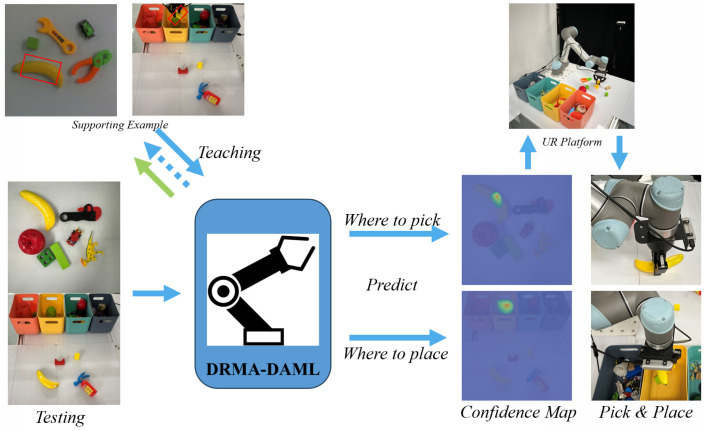
With the provision of a limited number of exemplars, our robotic apparatus is capable of promptly learning to grasp and position the object within the appropriate receptacle. The level of luminance serves as an indicator of the confidence rating.

Humans often engage in a process of gradually deepening their thoughts when reflecting on a particular matter, with effective decision making arising from the integration of careful deliberation and underlying intuitive insights. Drawing inspiration from both cognitive processes and biological learning mechanisms—where attention and memory retention play pivotal roles in adaptive behavior—we propose a novel framework termed Residual Multi-Attention Domain-Adaptive Meta-Learning (DRMA-DAML) (Figure 1). This framework leverages residual attention mechanisms to combine shallow and deep features, enhancing task processing by capturing both immediate and deeper contextual information for more efficient and adaptive learning.

In this work, our principal contributions are encapsulated as follows:

1. We have constructed a residual multi-attention network designed to reinforce the network’s retention of shallow features through an array of attention modules. Subsequently, we have augmented this network by integrating deformable convolutions, aiming to escalate model sophistication without a proportional increase in network depth or breadth.
2. We have curated a dataset for meta-learning tasks, consisting of a goal prediction network with 9600 human demonstrations and an equal number of robotic demonstrations. This dataset is uniquely tailored for meta-learning applications in robotic grasping, offering substantial support for classification-driven perception tasks within this domain. In contrast to prior studies, our dataset introduces pick-and-place experiments set against complex backgrounds, thereby validating the efficacy of deformable convolutions in managing intricate scenes.
3. Our enhanced model leverages both human and robotic demonstrations more effectively, achieving a higher success rate in comprehensive real-world experiments. This underscores the efficacy and robustness of DRMA-DAML, which notably surpasses the performance of state-of-the-art methodologies.

## 2. Related Works

### 2.1. Imitation Learning

Imitation learning, or learning from demonstrations (LfD), has emerged as a pivotal approach in enabling robots to acquire diverse skills and adapt to a broad spectrum of scenarios. This is often achieved by collecting demonstrations through teleoperation [20] or kinesthetic teaching [21], which serve as the foundation for teaching robots. However, many methods require meticulous specification of the correspondence between human behaviors and robot actions, or the manual definition of how to translate video demonstrations into executable robot actions (e.g., [22,23,24]).

In response to the challenges posed by imperfect or incomplete demonstrations, active learning strategies [25,26,27] have been explored to better capture human preferences. Yet, this approach is time-intensive as it necessitates a user-in-the-loop learning process, which can be cumbersome [28].

Building on previous successes where inverse reinforcement learning (IRL) has been integrated into imitation learning or LfD (e.g., [29,30,31,32]), ref. [33] showed that learning from failure—encompassing suboptimal and failed demonstrations—can lead to faster learning and better generalization compared to traditional IRL methods. However, IRL’s reliance on additional expert knowledge poses difficulties in learning and optimizing rewards for various unspecified or previously unencountered tasks [34].

While meta-learning has been extensively explored in few-shot object detection [35], multi-user preferences [36], no-reference image quality assessment [37], and image classification [38,39,40], few of these methods reliably enable robots to learn from visual or video demonstrations. Consequently, several representative meta-learning approaches (e.g., [9,11,12,16]) have been investigated for their potential in rapid adaptation to previously unseen environments through learning from visual or video demonstrations.

Given that traditional meta-learning methods, which involve deepening or widening neural networks, offer only marginal improvements in model performance, a novel framework—Multi-Level and Multi-Attention Domain-Adaptive Meta-Learning (MLMA-DAML)—was proposed in [19]. The MLMA-DAML framework has demonstrated that feature extraction through various attention heads at different levels and scales is crucial for enhancing meta-learning model performance.

Addressing these challenges, our model, DRMA-DAML, employs residual connections and a dynamic weighting mechanism that allows the model to focus on both the shallow and deep layers of the neural network, thereby significantly enhancing model output effectiveness. This approach not only refines the learning process but also underscores our commitment to advancing the frontiers of robotic learning through innovative meta-learning strategies.

### 2.2. Residual Attention Mechanism

In the field of neural networks, attention mechanisms are crucial for enhancing task performance. Residual attention mechanisms have received significant attention. For computer vision, Wang et al. [41] proposed a residual attention network with residual connections in attention modules for better hierarchical feature capture. Hu et al. [42] introduced the squeeze-and-excitation attention mechanism for channel-wise feature recalibration. For natural language processing, Vaswani et al. [43] proposed the self-attention mechanism, which is widely used in tasks like machine translation and text generation. Zhang et al. [44] proposed a residual temporal attention mechanism for video analysis. Li et al. [45] extended the residual attention mechanism to multi-modal data.

For artificial intelligence perception, Li et al. [46] proposed MANet, integrating kernel and channel attention mechanisms. By reducing the dot-product attention complexity to O(N) via kernel attention, MANet efficiently captures global contextual dependencies and enhances feature map discriminability, outperforming state-of-the-art methods.

These residual attention mechanisms have evolved and have been integrated into various neural network architectures, providing powerful tools to improve model performance and generalization in many applications.

### 2.3. Deformable Convolution

The development of convolutional neural networks (CNNs) has always focused on better adapting to different input data. Traditional convolution operations have fixed kernel sizes and shapes, which limit the model’s ability to handle complex data to some extent. To address this issue, deformable convolution was introduced by Dai et al. [47]. By adding additional offsets to the traditional convolution kernel, the kernel can adaptively adjust its position within a certain range, thus better capturing the geometric changes of the input features. This method has achieved significant results in tasks such as object detection and semantic segmentation.

Subsequently, Zhu et al. [48] further improved deformable convolution and proposed deformable convolutional networks (DCNs) version 2. This version not only considers the position offset of the convolution kernel but also introduces additional deformable parameters, such as the size and aspect ratio of the convolution kernel, further improving the flexibility and expressiveness of the model. In addition, many research efforts have been dedicated to improving and expanding the application of deformable convolution. For example, some studies combined deformable convolution with attention mechanisms to better focus on important feature regions [49]. Other studies applied deformable convolution to other fields, such as video analysis [50] and medical image analysis [51].

As an effective technique, deformable convolution provides new approaches to improving the performance and adaptability of CNNs and has been widely used and studied in fields such as computer vision.

## 3. Methods

### 3.1. Background: Model-Agnostic Meta-Learning

Model-agnostic meta-learning (MAML) [10] focuses on optimizing the weights θ of a model $f_{\theta }$ so that it can effectively adapt to new tasks using one or more gradient steps and a limited set of training data while avoiding overfitting. When a new task $\tau_{i}$ is drawn from the distribution p(τ), the model’s parameters θ are refined through gradient descent with an inner learning rate η as follows:

(1) $$\phi =\theta -\eta \nabla_{\theta }L\left(f_{\theta },\tau_{i}\right)$$

where ϕ represents the task-specific model parameters after a single gradient update, initialized from the general parameters θ. The update step is computed by subtracting the product of the inner learning rate η and the gradient of the loss function $L\left(f_{\theta },\tau_{i}\right)$ with respect to θ. $L\left(f_{\theta },\tau_{i}\right)$ quantifies the error of the model $f_{\theta }$ when performing on task $\tau_{i}$, and $\nabla_{\theta }$ indicates the gradient, capturing how the loss changes with respect to the model parameters. This update mechanism ensures that the model effectively learns task-specific information while retaining its generalization ability across multiple tasks.

During the outer loop of the training process, the adapted policy $f_{\phi }$ is assessed on query data from task $\tau_{i}$. The resulting performance on these query examples informs the optimization of the initial policy $f_{\phi }$, using a meta-learning rate β as follows:

(2) $$\theta \leftarrow \theta -\beta \nabla_{\theta }\sum L\left(f_{\phi },\tau_{i}\right)$$

### 3.2. DRMA-DAML

DRMA-DAML seeks to train a model θ capable of rapidly adapting to novel, unseen scenarios (query tasks) through gradient updates based on demonstrations (support tasks).

**Figure 2:**
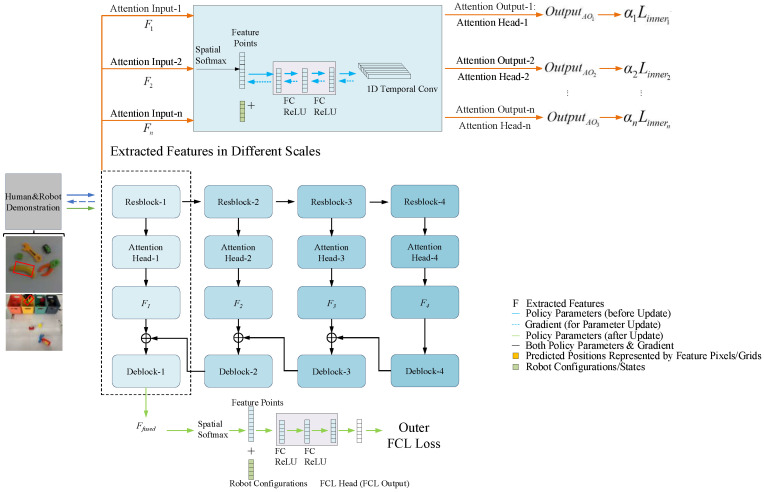
Illustration of DRMA-DAML. The DRMA-DAML framework begins by extracting a comprehensive set of features, denoted as $F_{1},F_{2},\cdots ,F_{n}$, encompassing both shallow and deep representations.

For simplicity, this work assumes a single gradient update for θ to derive the task-specific model ϕ for any given support task. As illustrated in Figure 2, the DRMA-DAML framework segments the meta-training process into two distinct phases: (1) the inner loop/update, which employs multiple attention heads, and (2) the outer loop/update, which leverages fully connected layer (FCL) heads.

#### 3.2.1. Internal Update in DRMA-DAML

Unlike MAML-DAML, which computes gradients across the entire network as a whole, DRMA-DAML calculates gradients independently for each block. This block-wise gradient computation allows for more fine-grained updates tailored to the specific features extracted by individual attention heads. Additionally, since the features output by different attention heads may vary in dimensions, a ‘deblock’ module is introduced to unify multi-scale features. The unified features are subsequently combined through residual summation and further processed to compute the final fully connected layer (FCL) loss.

The internal loss in DRMA-DAML is designed to adapt the model to each support task $D_{s}$, where the computation is distributed across multiple attention heads. Each attention head uses its assigned block to compute its contribution to the loss. For each attention head, the domain adaptation internal loss is defined as

(3) $$\begin{array}{cc} L_{{\mathrm{inner}}_{k}} & =\alpha_{k}{∥{Output}_{AO_{k}}∥}_{2}^{2}\\ \; & =\alpha_{k}{∥W_{k}f_{k}+b_{k}∥}_{2}^{2}, \end{array}$$

where $L_{{\mathrm{inner}}_{k}}$ represents the weighted loss for the *k*-th block, $\alpha_{k}$ denotes the importance factor of the block, $f_{k}$ is the input feature to the *k*-th attention head, and $W_{k}$ and $b_{k}$ are the corresponding weights and biases, respectively. The attention output $Output_{AO_{k}}$ is therefore expressed as $W_{k}f_{k}+b_{k}$.

The attention factor $\alpha_{k}$ determines the relative importance of the *k*-th attention head and is calculated using a predefined decay factor *d*. A smaller *d* assigns higher significance to earlier attention heads, while a larger *d* emphasizes later attention heads. For example, when n=3 and d=0.5, the resulting $\alpha_{1:n}$ values are [2.0,1.0,0.5], whereas n=5 and d=2 yield $\alpha_{1:n}=\left[0.25,0.5,1.0,2.0,4.0\right]$.

After obtaining the output from each attention head, the features are processed through the ‘deblock’ module to align the dimensions of multi-scale features. The aligned features are then aggregated via residual summation, ensuring that both shallow and deep feature representations are effectively integrated. These aggregated features are subsequently passed through spatial softmax, fully connected layers (FCLs), and a ReLU activation function to produce the final output, which is used to compute the FCL loss.

Once the individual $L_{{\mathrm{inner}}_{k}}$ values are computed and weighted by $\alpha_{k}$, the model parameters ϕ are updated by summing the gradients from all blocks and applying stochastic gradient descent (SGD):

(4) $$\phi =\theta -\eta \sum_{k=1}^{n}\nabla_{\theta }\left(\alpha_{k}L_{{\mathrm{inner}}_{k}}\right),$$

where η represents the learning rate for the inner loop. We usually set the value at 0.0001. This block-wise computation, combined with feature alignment and residual aggregation, enhances DRMA-DAML’s ability to adapt to multi-scale features and maintain critical shallow representations, improving meta-learning performance for downstream tasks.

In this equation, ϕ represents the updated model parameters after summing the contributions from all *n* blocks. Each block’s contribution is determined by the gradient of its weighted loss $\alpha_{k}L_{{\mathrm{inner}}_{k}}$ with respect to θ, where $\alpha_{k}$ is the importance factor of the *k*-th block. The learning rate η, set to 0.0001 by default, controls the step size of this stochastic gradient descent (SGD) update. This approach ensures that information from both shallow and deep features is effectively aggregated, enabling the model to adapt to multi-scale features and retain critical shallow representations.

#### 3.2.2. Outer Update of DRMA-DAML

Following the inner loop, where the task-specific model parameters ϕ are obtained, the meta-performance is optimized in the outer loop using the query task $D_{r_{i}}^{q}$ as follows:

(5) $$\begin{array}{cc} \min_{\theta }L_{FCL}\left(f_{\phi },D_{r_{i}}^{q}\right) & =\min_{\theta }L_{FCL}\left(f_{\theta -\eta \nabla_{\theta }\sum L_{\mathrm{inner}}\left(f_{\theta },D^{s}\right)},D_{r_{i}}^{q}\right)\\ \; & =\min_{\theta }{∥O_{FCL}\left(f_{\phi },D_{r_{i}}^{q}\right)-a∥}_{2}^{2} \end{array}$$

Here, $O_{FCL}\left(f_{\phi },D_{r_{i}}^{q}\right)$ represents the output of the fully connected layer (FCL) head given the model parameters ϕ and the query task $D_{r_{i}}^{q}$, while *a* denotes the corresponding supervised robotic action. The term $\min_{\theta }$ indicates that the optimization process seeks to find the parameters θ that minimize the outer loss $L_{FCL}$.

In the outer update stage, the Adam optimizer (adaptive moment estimation) is employed to adjust the model parameters by minimizing the outer loss $L_{FCL}\left(f_{\phi },D_{r_{i}}^{q}\right)$. Unless otherwise specified, the default learning rate for the outer loop is set to 0.0001.

**Figure 3:**
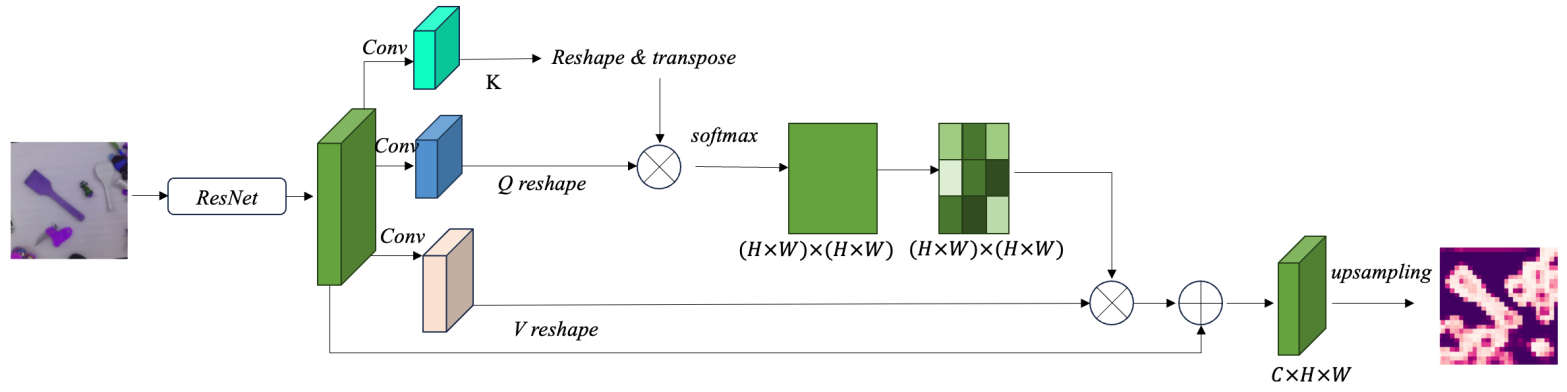
The process of feature extraction using an attention mechanism. The input feature map, extracted by the ResNet backbone, is processed through three convolutional layers to generate the query *Q*), key (*K*), and value (*V*) feature matrices. The key matrix is reshaped and transposed, while the query matrix is reshaped. These are then used to compute the attention score matrix via dot product. The scores are normalized using the softmax function to produce the attention weight matrix. Subsequently, these weights are applied to the value matrix through weighted summation, resulting in an enhanced feature representation. Finally, the enhanced features are combined with the input features using a residual connection, followed by an upsampling operation to produce the output.

The attention mechanism computes the value (*V*) as follows. In Figure 3, Given an input feature matrix $X\in R^{N\times C}$, where N=H×W represents the spatial dimension and *C* denotes the number of channels, the query (*Q*), key (*K*), and value (*V*) matrices are obtained by applying learnable weight projections: $Q=XW_{q}\in R^{N\times D_{k}}$, $K=XW_{k}\in R^{N\times D_{k}}$, and $V=XW_{v}\in R^{N\times D_{v}}$, where $D_{k}$ and $D_{v}$ are the respective dimensions. The standard dot-product attention mechanism is then computed as $D\left(Q,K,V\right)=\rho \left(QK^{T}\right)V$, where $\rho \left(QK^{T}\right)=\mathrm{softmax}\left(QK^{T}\right)$. However, this approach incurs a computational complexity of $O(N^{2})$, making it inefficient for large-scale inputs. To address this issue, this paper proposes a Kernel Attention Mechanism (KAM), which replaces softmax with a kernel smoother:

(6) $$D{\left(Q,K,V\right)}_{i}=\frac{\sum_{j=1}^{N}\mathrm{softplus}{\left(Q_{i}\right)}^{T}\mathrm{softplus}\left(K_{j}\right)V_{j}}{\sum_{j=1}^{N}\mathrm{softplus}{\left(Q_{i}\right)}^{T}\mathrm{softplus}\left(K_{j}\right)},$$

where the softplus function is defined as $\mathrm{softplus}\left(x\right)=\log\left(1+e^{x}\right)$. This formulation allows for a more computationally efficient implementation, reducing the complexity to O(N). Finally, a residual connection is added to preserve the original input features, leading to the final output O=D(Q,K,V)+X. By leveraging kernel-based attention, the proposed method significantly improves efficiency while maintaining the ability to capture long-range dependencies in feature representations.

### 3.3. DRMA-DAML++

**Figure 4:**
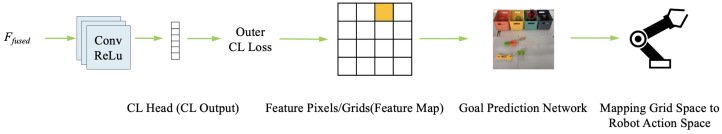
Structural schematic diagram of DRMA-DAML++. It incorporates a goal prediction network in the outer loop, which corresponds to the convolutional layer (CL) head and is based on DRMA-DAML. This enhancement aims to improve the model’s robustness in handling position shifts. By doing so, DRMA-DAML++ can better adapt to the requirements of various tasks across different scenarios and enhance the model’s performance and accuracy. The yellow box marks the region where the model’s performance is most affected by positional shifts.

**Figure 5:**
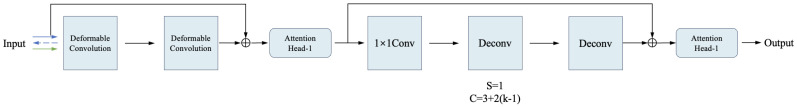
Structural schematic diagram of the block, which is framed by the dashed box in Figure 2, a component in both DRMA-DAML and DRMA-DAML++. The block is designed to extract features at different scales. Each block consists of two deformable convolutional layers with specific kernels and a one-dimensional convolutional layer. Through the combined operations of these layers, the block can effectively extract feature information from the input data. This feature information is crucial for subsequent task processing and decision making, enabling the model to better understand and process the input data.

Recognizing that predicting positions in the feature/pixel space (refer to Figure 4) demonstrates greater robustness to position shifts, we introduce a goal prediction network in the outer loop of DRMA-DAML. This addition corresponds to the CL (convolutional layer) head. Consequently, the outer loss in DRMA-DAML++ is formulated as

(7) $$\begin{array}{cc} \; & L_{\mathrm{DRMA}-\mathrm{DAML}++}\left(f_{\left(\phi ,F_{\mathrm{fused}}\right)},D_{r_{i}}^{q}\right)=\\ & L_{\mathrm{FCL}}\left(f_{\phi },D_{r_{i}}^{q}\right)+L_{\mathrm{CL}}\left(f_{\left(\phi ,F_{\mathrm{fused}}\right)},D_{r_{i}}^{q}\right), \end{array}$$

where $L_{\mathrm{DRMA}-\mathrm{DAML}++}$ represents the total outer-loop loss in DRMA-DAML++, comprising two components: the fully connected layer (FCL) loss, $L_{\mathrm{FCL}}$, and the convolutional layer (CL) loss, $L_{\mathrm{CL}}$. The FCL loss, $L_{\mathrm{FCL}}\left(f_{\phi },D_{r_{i}}^{q}\right)$, evaluates the global semantic accuracy of the model’s predictions for the query task $D_{r_{i}}^{q}$. Meanwhile, the CL loss, $L_{\mathrm{CL}}\left(f_{\left(\phi ,F_{\mathrm{fused}}\right)},D_{r_{i}}^{q}\right)$, focuses on aligning the predicted and ground-truth feature maps at the pixel or grid level, enhancing spatial precision. Here, $F_{\mathrm{fused}}=\sum_{k=1}^{n}\alpha_{k}F_{k}$ represents the fused features, combining multi-scale information from multiple attention heads weighted by importance factors $\alpha_{k}$. By integrating these two losses, DRMA-DAML++ ensures robust learning of both global representations and fine-grained spatial details, improving its adaptability and generalization across diverse tasks.

(8) $$F_{\mathrm{fused}}=\sum_{k=1}^{n}\alpha_{k}F_{k},$$

where $F_{\mathrm{fused}}$ represents the fused extracted features, which are subsequently utilized to compute the feature map depicted in Figure 5.

Similar to $L_{\mathrm{FCL}}\left(f_{\phi },D_{r_{i}}^{q}\right)$, as defined in DRMA-DAML, the term $L_{\mathrm{CL}}\left(f_{\left(\phi ,F_{\mathrm{fused}}\right)},D_{r_{i}}^{q}\right)$ is given by

(9) $$L_{\mathrm{CL}}\left(f_{\left(\phi ,F_{\mathrm{fused}}\right)},D_{r_{i}}^{q}\right)={∥O_{\mathrm{CL}}\left(f_{\left(\phi ,F_{\mathrm{fused}}\right)},D_{r_{i}}^{q}\right)-m∥}_{2}^{2},$$

where $O_{\mathrm{CL}}\left(f_{\left(\phi ,F_{\mathrm{fused}}\right)},D_{r_{i}}^{q}\right)$ represents the output of the outer CL head, which operates on the query task $D_{r_{i}}^{q}$ and the fused feature representation $F_{\mathrm{fused}}$. The variable *m* corresponds to the supervised feature map derived from $D_{r_{i}}^{q}$, serving as the ground truth for training and evaluation purposes.

In DRMA-DAML++, the feature map is assumed to have dimensions M×M, consisting of M×M grids or pixels to encapsulate task-specific spatial information. This design facilitates more robust representation learning for diverse tasks. Consistent with previous research, we configure M=8 as the default size in this study, balancing computational efficiency and feature resolution.

### 3.4. Deformable Convolution

In traditional convolutional neural networks (CNNs), the convolution operation is performed through dot-product accumulation within a sliding window. Given an input feature map *f* and a convolution kernel *w*, the standard convolution operation can be expressed as

(10) $$y\left(p_{0}\right)=\sum_{p_{n}\in R}w\left(p_{n}\right)\cdot f\left(p_{0}+p_{n}\right)$$

Here, $y(p_{0})$ represents the value at position $p_{0}$ on the output feature map, R denotes the area covered by the convolution kernel, and $p_{n}$ is the position offset relative to $p_{0}$.

Deformable convolution enhances the flexibility of convolution operations by introducing additional learnable offsets $\Delta p_{n}$, enabling the convolution kernel to adaptively adjust its shape to better capture relevant information. This operation can be formulated as

(11) $$y\left(p_{0}\right)=\sum_{p_{n}\in R}w\left(p_{n}\right)\cdot f\left(p_{0}+p_{n}+\Delta p_{n}\right)$$

In this equation, $\Delta p_{n}$ is an offset learned through an additional network layer, which allows the convolution kernel to dynamically adjust its sampling positions on the input feature map.

Given that the offset sampling points may not exactly align with the grid points of the input feature map, deformable convolution employs bilinear interpolation to compute the feature values at these sampling points. For any real-valued position $p^{\prime }$, its feature value can be derived from the values of the surrounding four integer grid points, as expressed by

(12) $$f\left(p^{\prime }\right)=\sum_{q}G\left(q,p^{\prime }\right)\cdot f\left(q\right)$$

Here, *q* traverses the four integer positions surrounding $p^{\prime }$, and *G* is a bilinear interpolation function that computes the weight based on the relative position relationship between *q* and $p^{\prime }$. The bilinear interpolation kernel *G* can be decomposed into two one-dimensional kernels as follows:

(13) $$G\left(q,p\right)=g\left(q_{x},p_{x}\right)\cdot g\left(q_{y},p_{y}\right)$$

(14) g(a,b)=max(0,1−|a−b|)

The function g(a,b) defines a one-dimensional linear interpolation kernel that calculates the weight based on the distance between two points, *a* and *b*. Specifically, |a−b| represents their absolute distance, and the weight is computed as 1−|a−b|, where smaller distances result in higher weights. The max(0,·) operation ensures non-negative weights by setting the weight to zero when |a−b|>1. This constraint ensures that the kernel has a finite support range of [a−1,a+1], restricting non-zero weights to nearby points. Such a design not only captures local spatial relationships but also ensures computational efficiency by focusing on a small number of relevant neighbors.

This effectively means that G(q,p) is non-zero for only a few neighboring locations *q*, allowing the interpolation to be performed using a limited number of operations and ensuring computational efficiency.

The offsets $\Delta p_{n}$ are learned by applying a convolutional layer over the input feature map. This additional convolutional layer has the same spatial resolution and dilation as the deformable convolution layer it supports. The output of this layer produces the offset fields, which have the same spatial resolution as the input feature map, but with a channel dimension corresponding to the 2D offsets for each position in the grid R. These offset fields are learned simultaneously with the convolutional kernels during training through standard backpropagation.

## 4. Experiments

Sorting parcels or waste is a critical task in both everyday life and industrial processes. This study explores how robots can be guided to the appropriate container or bin for an object based on visual demonstrations. In line with prior research, we presume that the UR5 robotic arm can grasp an object under the guidance of a trained neural model or through the ROS interface and subsequently place the object into a specified container or bin.

### 4.1. Dataset

**Figure 6:**
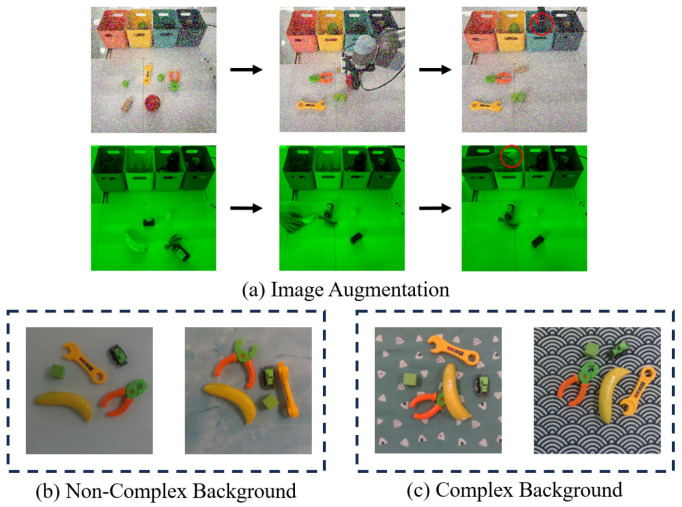
(**a**) Examples of image augmentation. The red circles indicate the target boxes/bins where the objects need to be placed. (**b**,**c**) Scene settings in non-complex and complex backgrounds, respectively.

As depicted in Figure 1, a pair of RealSense D435 cameras were utilized to gather real-world data for training and testing purposes. These cameras were operated through a computer interface that facilitated interaction with the images using a mouse, and the collected data were subsequently expanded using image enhancement techniques (Figure 6), including random image noise, color transformations, and border cropping. The random noise applied to images included Gaussian noise, salt-and-pepper noise, and other random variations introduced during preprocessing. Before being fed into the model, all images were resized to a standardized resolution of 256×256 pixels to ensure consistency and compatibility with the model’s input requirements. Our training dataset comprised 9600 human demonstration videos (for support tasks) and 9600 robotic demonstration videos (for query tasks), organized into approximately 1920 tasks with distinct goals or scenarios, each represented by five demonstrations or videos. While TecNets [12] required only two keyframes (the initial and final states) to guide the robot in placement tasks, our experiments for DRMA-DAML/DRMA-DAML++ collected demonstrations or videos containing three keyframes. In this context, three keyframes for a demonstration or video were deemed sufficient and convenient for teaching the robot about placement tasks. To assess effectiveness and robustness, we evaluated our methods across three different settings:

1. A test set (Test Set 1) that included real-world tasks with 160 support tasks (five human demonstrations or videos per task) and 160 query tasks (five robotic scenarios per task).
2. A test set (Test Set 2) that encompassed 3200 support tasks (five human demonstrations or videos per task) and 3200 query tasks (five robotic scenarios per task). This set was significantly more challenging, as it incorporated image enhancement techniques applied to Test Set 1.
3. A test set (Test Set 3) that consisted of 3200 support tasks (five robotic demonstrations or videos per task) and 3200 query tasks (five robotic scenarios per task).

It is important to note that the aim of Test Set 2 and Test Set 3 was to evaluate the model’s robustness under various conditions, including new objects, background colors, and even image noise. Objects and bins were randomly arranged on a table, accompanied by six to ten distractors, such as bins of different colors and objects.

### 4.2. Task Definition

A pick-and-place task is illustrated in Figure 1. A placement task was deemed successful if the robot placed the grasped object into the designated box or bin as indicated by the human demonstration. For ease of definition, tasks are primarily identified by the color of the bins, and it has been shown in both simulated and real-world environments that MAML-based or meta-learning methods can distinguish not only between bins of different colors but also objects of varying shapes, sizes, and textures placed in diverse positions. To measure accuracy, a task was considered successful if the predicted end-effector position was within the boundaries of the designated box or bin.

### 4.3. Model Configuration

The number of convolutional kernels for each convolutional layer was set to 64 by default, except for the final convolutional layer in the goal prediction network, which was configured with a single kernel. Fully connected layers were set to a size of 200, and each block consisted of two deformable convolutional layers with 32 kernels of size 3×3 to dynamically capture spatial features, followed by one 1D convolutional layer with 32 kernels of size 1×1 for feature integration and channel-wise transformations. Unless otherwise specified, the stride size was set to 1 for all convolutional layers. Layer normalization was employed consistently to standardize the features extracted by the network, enhancing convergence and stability during training.

During the training phase, the models utilized 35.8 M trainable parameters and underwent 30,000 iterations with a meta-batch size of 16. After training, the model was evaluated in testing scenarios to predict the end-effector position for previously unseen environments or tasks. The methods were evaluated under various random seeds (e.g., 0, 1, and 2), and the best-performing results were reported.

### 4.4. Experimental Results and Analysis

**Figure 7:**
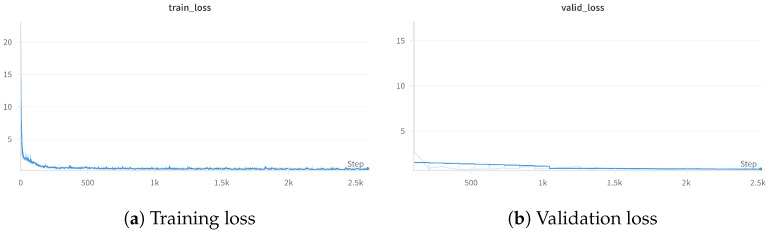
Training and validation losses during the training process. The training loss decreases rapidly in the initial steps and stabilizes as training progresses, showing effective optimization. The validation loss follows a similar trend and eventually converges to a stable low value, indicating good generalization performance without significant overfitting.

We primarily compared our proposed methods with the most relevant approaches, including DAML, TaR-MIL, MLMA-DAML/MLMA-DAML++, and RDAML. Given that MLMA-DAML++ demonstrates superior performance compared to MLMA-DAML, our proposed DRMA-DAML and DRMA-DAML++ methods are predominantly implemented based on the MLMA-DAML++ framework. In this section, we present and analyze our experimental results under three distinct testing settings. The Figure 7 illustrates our training process, where the convergence of the loss function demonstrates the effectiveness of the model.

As summarized in Table 1, three configurations based on DAML were employed in [19]: (1) a relatively shallow network with five convolutional layers, where each layer comprised 64 convolutional kernels of size 3×3, referred to as DAML; (2) a more complex network with 12 convolutional layers, each containing 64 convolutional kernels of size 3×3, denoted as DAML${\,}^{†}$; and (3) a deeper network with 18 convolutional layers, each with 64 convolutional kernels of size 3×3, referred to as DAML${\,}^{*}$.

As anticipated, DAML${\,}^{†}$ and DAML${\,}^{*}$ exhibited inferior performance compared to DAML. This observation highlights that simply increasing the depth of the network does not necessarily enhance meta-learning performance. Two primary reasons account for this result: (1) deeper networks are more susceptible to overfitting, and (2) shallow features, which are critical for the adaptation process in meta-learning, tend to be overlooked or diminished in deeper architectures.

It is important to note that employing a fixed convolutional kernel size of 3×3 in a deeper network yields limited improvements in model performance. Consequently, our proposed methods adopt models with multiple convolutional kernel sizes to enhance the meta-learning capability, facilitating adaptation to features of varying scales.

Following previous work, we first conducted an extensive study on DRMA-DAML under different attention head sizes *n* and decay factors *d*. Under the settings where human demonstrations were used as support tasks to train and test the model on Test Set 1 and Test Set 2, the best results of our proposed DRMA-DAML demonstrated that it outperformed most related state-of-the-art methods, as shown in Table 1 and Table 2. Experimental results confirm that the deformable residual multi-attention mechanism we introduced improves the model’s capability to focus on shallow data features compared to previous models.

At the same time, experiments conducted on complex backgrounds demonstrated that our model achieved a higher success rate compared to previous works. DRMA-DAML${\,}^{†}$ represents the ablation experiment of the model without deformable convolution. Ablation studies indicated that deformable convolutions effectively managed grasping and placing tasks in intricate environments. These findings underscore the superiority of deformable convolutions in extracting object features in complex backgrounds.

Unlike MLMA-DAML and MLMA-DAML++, which achieved the best results with d=0.5, our proposed DRMA-DAML performed the best when d=1.0 because the decay factor *d* had different optimal values as the model structure changed. The proposed framework obtained the best results with n=5, while the success rates dropped as the model deepened, as shown in Table 1 and Table 2. This further verifies that simply constructing a highly complex model is not the best for meta-learning. As the model becomes more complex, it tends to overfit the training set, and its generalization ability worsens on the testing set.

Subsequently, we present a comprehensive analysis of how the model parameters and settings influenced the overall performance of the DRMA-DAML framework. By exploring the distinctions between the FCL head and CL head, as well as the effects of key parameters such as the number of attention heads (*n*) and the decay factor (*d*), we identified the critical factors underlying the model’s superior performance. We further studied the effect of background complexity to offer more insights into the robustness and adaptability of the proposed framework to diverse task conditions.

The experimental results indicated that the CL head usually achieved superior performance compared to the FCL head under complex backgrounds, mainly due to the ability of the CL head to attend to fine-grained spatial features. Specifically, in Test Set 1, the successful sub-detection ratios of the CL head under complex backgrounds were 95.34% for DRMA-DAML++ and 94.76% for DRMA-DAML, but these ratios were only 94.12% and 94.09% for the FCL head. A similar outcome was observed in Test Set 2, where the CL head performed at 90.34%, slightly better than the 89.01% attained by the FCL head. This performance gap further suggests that a CL head can directly serve spatial feature maps, making it more applicable to tasks that require accurate localization from noisy or distracting images. In contrast, the FCL head is better suited for tasks that rely more on global semantic abstractions, as it shows strong performance in non-complex backgrounds. The superiority of the CL head in complex environments underscores its robustness in handling noisy inputs and maintaining accuracy in challenging scenarios.

The number of attention heads (*n*) significantly affects the model’s ability to capture multi-scale features. The experimental results show that *n* = 5 yielded the best performance. For instance, in Test Set 1, the success rates of DRMA-DAML with *n* = 5 reached 96.93% and 94.76%, whereas they dropped to 92.45% and 91.01% with *n* = 3, and to 95.21% and 92.34% with *n* = 7. A lower number of attention heads, such as *n* = 3, may limit the model’s capacity to capture diverse feature scales, particularly in complex backgrounds where shallow and deep features must be effectively fused. In contrast, too many heads (e.g., *n* = 7) increase model complexity, making training more difficult and leading to overfitting, thus reducing generalization performance. The optimal setting of *n* = 5 strikes a balance by achieving sufficient feature extraction across scales while maintaining manageable model complexity and robust generalization.

The decay factor (*d*) determines the relative weighting of shallow and deep attention heads, and its configuration plays a pivotal role in performance. The experimental findings indicate that *d* = 1.0 offered the best balance, yielding superior results across tasks. For example, in Test Set 1, under complex backgrounds, the success rate with *d* = 1.0 was 94.76%, compared to 90.32% with *d* = 0.1 and 93.61% with *d* = 10.0. When *d* was too small, such as 0.1, the model overemphasized shallow features, potentially neglecting deep semantic information critical for overall task performance. Conversely, a large value of *d*, such as 10.0, placed excessive weight on deep features, leading to the loss of essential spatial details that are particularly important in complex backgrounds. Thus, *d* = 1.0 achieved an optimal balance, enabling effective collaboration between shallow and deep features, resulting in superior performance under both non-complex and complex scenarios. The background complexity significantly affected the experimental outcomes, with models performing better in non-complex backgrounds.

In Test Set 1, DRMA-DAML achieved a success rate of 96.93% in non-complex backgrounds, while the rate dropped to 94.76% in complex backgrounds. Non-complex backgrounds contain fewer distractors, allowing the model to rely predominantly on deep features for task completion. Similarly under complex backgrounds in Test Set 2, the success rate with *d* = 1.0 reached 90.25%, compared to 83.35% with *d* = 0.1 and 85.49% with *d* = 10.0. When *d* was too small, such as 0.1, the model placed excessive emphasis on shallow features, potentially neglecting critical deep semantic information essential for complex task execution. In contrast, complex backgrounds introduced significant challenges due to the presence of distractors and visual clutter, necessitating effective integration of shallow features (e.g., edges and contours) and deep features (e.g., semantic information) for precise localization and manipulation. DRMA-DAML addressed this challenge through its residual multi-attention mechanism and deformable convolutions, which dynamically adjusted sampling positions to adapt to spatial variations. These capabilities enabled the model to maintain robust performance in complex environments, highlighting its adaptability and effectiveness in diverse scenarios.

Given that humans can learn from demonstrations across different domains, an exploration of DRMA-DAML/DRMA-DAML++ was conducted in [19]. This exploration involved utilizing human demonstrations as support tasks during training to teach the robot and subsequently employing robotic demonstrations as support tasks during testing. As shown in Table 3, DRMA-DAML and DRMA-DAML++ demonstrated improvements in this scenario, indicating their potential in handling cross-domain tasks. However, due to the substantial differences between human and robotic demonstrations, such as variations in perspectives and morphologies, their performance remains limited.

Building on this challenge, Random Domain-Adaptive Meta-Learning (RDAML) was proposed [17] to address these limitations. Unlike previous methods that primarily relied on either human or robotic demonstrations as support tasks, RDAML facilitates joint learning from both human and robotic demonstrations, effectively bridging the domain gap and enhancing adaptation performance.

Inspired by the advancements of RDAML, we further explored the multi-domain learning capability of DRMA-DAML++ by combining it with RDAML. As shown in Table 4, our approach utilized both human and robotic demonstrations as support tasks during the training phase of DRMA-DAML++. During testing, DRMA-DAML++ was evaluated separately by learning from human demonstrations in Test Set 2 and robotic demonstrations in Test Set 3. This integration demonstrates that DRMA-DAML++ can effectively adapt to diverse domains, highlighting its robustness in multi-domain learning scenarios.

Given that RDAML has demonstrated that training the model with two randomly sampled domain-adaptive parameters a and b performs better than training the model with two fixed domain-adaptive parameters, a and b (e.g., a=b=1), the domain-adaptive parameters, a and b, in our proposed DRMA-DAML++ were randomly sampled in the range of [0,1] for convenience. To our surprise, we achieved the best success rate of 90.32% in Test Set 2 and the best success rate of 99.67% in Test Set 3, significantly higher than the success rates of RDAML.

As the goal of DRMA-DAML++ is to adapt to both human and robotic demonstrations well, we observed that there was a performance sacrifice in learning from human demonstrations, but significant performance gains in learning from robotic demonstrations. Thus, we considered averaging the success rates in Test Set 2 and Test Set 3 to be a more suitable evaluation of the model’s performance. In this way, we obtained an average success rate of 95.00%, 18.71% higher than RDAML.

These results demonstrate that our proposed method has a better generalization capacity to multiple learning domains.

## 5. Conclusions

In this paper, we propose a novel DRMA-DAML framework that enables robots to effectively learn from visual demonstrations by capturing and integrating features at different scales and levels. This framework leverages residual multi-attention mechanisms and deformable convolutions to achieve a critical balance between shallow and deep feature extraction. These components are pivotal in retaining spatial details and extracting high-level semantic information, which enhances the framework’s robustness in handling diverse and challenging scenarios. The integration of these mechanisms allows DRMA-DAML to excel in spatially complex environments, where the CL head demonstrates significant superiority over alternative configurations. Additionally, the optimal settings of the attention heads (*n*) and decay factor (*d*) further strengthen the framework’s reliability and adaptability.

Extensive experiments validate the effectiveness of DRMA-DAML and its enhanced variant, DRMA-DAML++, across various robotic manipulation tasks. These results indicate that both models consistently outperform state-of-the-art approaches under challenging conditions. The framework not only improves overall task performance but also offers a robust and flexible solution for rapid adaptation in robotic applications. The ability to handle complex visual environments with distractors and clutter makes it a valuable contribution to the field of meta-learning and robot learning from demonstrations.

Although this study primarily focuses on robotic manipulation tasks, the principles and techniques introduced in DRMA-DAML can be extended to other meta-learning tasks. Examples include image classification, object detection, and image segmentation, where learning from diverse features at different levels is equally critical. This generalizability underscores the potential of DRMA-DAML as a versatile tool for broader applications in meta-learning. Moving forward, we aim to expand this work to more complex and dynamic scenarios, exploring the boundaries of robotic learning and further contributing to advancements in both robotics and machine learning.

## 6. Discussion

The experimental results presented in the tables in this paper demonstrate that the integration of residual networks and deformable convolutions within the DRMA-DAML framework is critical for its superior performance in robotic grasping and placement tasks. These two components effectively address the challenges of feature retention, adaptability, and robustness, particularly in complex environments.

**Figure 8:**
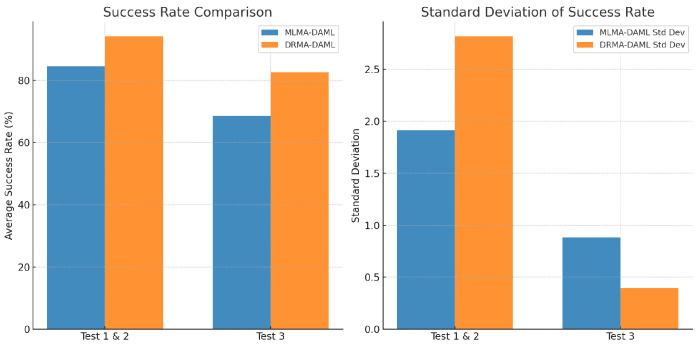
Comparison of MLMA-DAML and DRMA-DAML in terms of success rate and standard deviation. The left chart shows the average success rate, while the right chart presents the standard deviation, demonstrating the consistency of each model.

The results in Figure 8 indicate that DRMA-DAML outperforms MLMA-DAML, achieving a higher mean success rate (88.34% vs. 81.86%) with an average improvement of 6.48%. This demonstrates the effectiveness of residual multi-attention and deformable convolutions in enhancing feature extraction and generalization, particularly in complex environments. Additionally, DRMA-DAML exhibits greater stability with a lower average standard deviation (3.79% vs. 5.93%), resulting in a 2.14% reduction in performance variance. This suggests improved consistency, reduced overfitting, and better adaptability to environmental variations, reinforcing its reliability in real-world robotic learning tasks.

The *t*-test results in Table 5, with all *p*-values below 0.05, confirm that these improvements are statistically significant rather than due to random variations. The consistent statistical advantage across test sets reinforces DRMA-DAML’s effectiveness in both accuracy and robustness, making it a more reliable framework for meta-learning in robotic manipulation.

Residual connections play a pivotal role in mitigating the loss of shallow feature information as the network depth increases. Traditional deep neural networks often suffer from vanishing gradients and the gradual neglect of low-level spatial features, which are essential for precise object localization and manipulation. Residual connections alleviate these issues by enabling direct pathways for gradients to flow through the network during backpropagation, ensuring that both shallow and deep features are effectively utilized.

**Figure 9:**
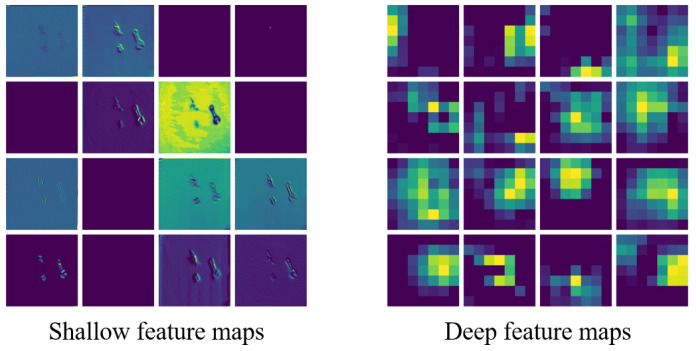
Comparison of shallow and deep feature maps in the DRMA-DAML framework. The left panel highlights shallow feature maps that preserve fine-grained spatial details, such as edges, textures, and contours, which are critical for precise object localization and recognition in complex scenes. The right panel demonstrates deep feature maps focusing on higher-level semantic abstractions, capturing broader contextual relationships but potentially losing spatial precision. Balancing shallow and deep features enhances the framework’s adaptability and robustness across diverse environments.

Shallow feature maps, as shown in Figure 9, preserve fine-grained spatial information such as edges, contours, and low-level textures. These features are crucial for tasks requiring precise localization and object recognition, especially in cluttered or visually complex scenes. The DRMA-DAML model, by incorporating residual multi-attention mechanisms, ensures that shallow features are retained throughout the network, leading to significantly higher success rates in both non-complex and complex backgrounds (e.g., Test Set 1 and Test Set 2). For instance, as reported in Table 1, DRMA-DAML achieves a success rate of 94.76% in complex backgrounds with d=0.5 compared to MAML-DAML’s 76.27%. This underscores the importance of shallow features in scenarios requiring precise object placement.

Deep feature maps, on the other hand, capture abstract and high-level semantic information, aiding in understanding complex object relationships and higher-level reasoning. However, excessive reliance on deep features can result in the loss of critical spatial details, adversely affecting performance in tasks requiring precision. The inclusion of deformable convolutions in DRMA-DAML mitigates this issue by dynamically adjusting sampling positions, thereby enhancing the spatial adaptability of deep features. By balancing shallow and deep features, DRMA-DAML maintains robustness and accuracy across diverse scenarios.

Deformable convolutions significantly enhance the network’s adaptability to spatial variations in input data, including object transformations, occlusions, and background clutter. Unlike standard convolutional layers with fixed receptive fields, deformable convolutions introduce learnable offsets that dynamically adjust the sampling grid, allowing the model to focus on the most relevant regions of the feature maps. This flexibility enables the network to effectively handle geometric distortions and varying object orientations, as demonstrated in Test Set 2, where DRMA-DAML achieves a 90.25% success rate in scenarios with complex backgrounds and random noise (Table 2). Moreover, deformable convolutions facilitate improved interaction between shallow and deep features by dynamically sampling from regions of interest, leading to better integration of low-level spatial information with high-level semantic abstractions—an essential capability for tasks requiring precise object recognition and manipulation. The importance of these layers is further underscored by ablation studies, when deformable convolutions are removed (DRMA-DAML^†^), the success rate in complex environments drops from 90.25% to 85.76% (Table 2), highlighting their critical role in managing feature extraction within spatially complex scenarios.

The experimental results reveal several key insights into the performance and effectiveness of the DRMA-DAML framework, highlighting its advantages over baseline models. These observations underscore the critical factors contributing to the framework’s success in robotic grasping and placement tasks:

1. Importance of Attention Mechanisms: The weighted attention factors assigned to shallow and deep features play a pivotal role in balancing their contributions. The results show that models with optimally tuned decay factors (*d* = 1.0 for DRMA-DAML) achieve the best performance, as shallow features are effectively emphasized without compromising the abstraction power of deeper layers.
2. Model Complexity and Overfitting: Increasing the depth of the network, as in DAML† and DAML*, does not lead to better performance. Instead, these deeper models exhibit signs of overfitting and diminished generalization ability. This highlights the importance of retaining shallow features rather than blindly deepening the network.
3. Robustness in Complex Backgrounds: DRMA-DAML’s superior performance in complex environments (e.g., random noise and distractors) demonstrates its robustness. This robustness is attributed to the deformable convolution layers, which adaptively extract features from spatially transformed inputs, preserving critical shallow and deep feature interactions.
4. Multi-Domain Generalization: The experiments on Test Set 2 and Test Set 3, involving human and robotic demonstrations as training data, further validate the framework’s adaptability across domains. DRMA-DAML++ achieves an average success rate of 95.00% (Table 4), outperforming RDAML by 18.71%. This result emphasizes the framework’s ability to generalize by leveraging both shallow and deep features across diverse input domains.

Future research could explore optimizing the trade-off between shallow and deep features using learnable attention-weighting mechanisms. Investigating alternative attention architectures or hybrid models could further enhance the interplay between shallow and deep features for complex tasks. In conclusion, these results highlight the critical role of shallow feature retention, balanced with deep feature abstraction, in achieving state-of-the-art performance in robotic grasping tasks. DRMA-DAML’s innovative use of residual multi-attention mechanisms and deformable convolutions sets a new benchmark for learning from demonstrations in dynamic and complex environments.

## Figures and Tables

**Table 1:** Using human demonstrations as support tasks to train and test the model in Test Set 1. † denotes the ablation study, representing the model without deformable convolutions. N indicates a pick-and-place task on a non-complex background. C indicates a pick-and-place task on a complex background. ‘${\,}_{F}$’ and ‘${\,}_{C}$’ indicate the test results on the FCL head and CL head, respectively.

Method	Hyperparameters		N	C
	*n*	*d*		
DAML [15,19]	-	-	80.26%	74.65%
TaR-MIL [16,19]	-	-	70.84%	62.38%
DAML${\,}^{†}$ [19]	-	-	74.46%	67.89%
DAML [19]	-	-	72.17%	66.19%
MLMA-DAML [19]	3	0.5	83.56%	76.27%
MLMA-DAML [19]	5	0.5	82.38%	75.84%
MLMA-DAML${\,}_{++F}$ [19]	7	0.5	85.83%	83.12%
MLMA-DAML${\,}_{++C}$ [19]	7	0.5	86.46%	84.40%
DRMA-DAML${\,}_{F}^{†}$ [19]	5	1.0	90.52%	84.53%
DRMA-DAML${\,}_{C}^{†}$ [19]	5	1.0	91.23%	84.72%
DRMA-DAML (ours)	3	0.1	91.54%	90.43%
DRMA-DAML (ours)	3	0.5	93.64%	90.57%
DRMA-DAML (ours)	3	1.0	92.45%	91.01%
DRMA-DAML (ours)	3	2.0	94.63%	91.25%
DRMA-DAML (ours)	3	10.0	93.76%	90.37%
DRMA-DAML${\,}_{F}$ (ours)	5	0.1	92.17%	90.33%
DRMA-DAML${\,}_{C}$ (ours)	5	0.1	92.23%	90.32%
DRMA-DAML${\,}_{F}$ (ours)	5	0.5	92.36%	92.00%
DRMA-DAML${\,}_{C}$ (ours)	5	0.5	93.13%	92.55%
DRMA-DAML${\,}_{F}$ (ours)	5	1.0	95.96%	94.09%
DRMA-DAML${\,}_{C}$ (ours)	5	1.0	**96.93**%	**94.76**%
DRMA-DAML${\,}_{F}$ (ours)	5	2.0	95.78%	94.14%
DRMA-DAML${\,}_{C}$ (ours)	5	2.0	95.86%	94.19%
DRMA-DAML${\,}_{F}$ (ours)	5	10.0	93.33%	93.90%
DRMA-DAML${\,}_{C}$ (ours)	5	10.0	95.46%	93.61%
DRMA-DAML (ours)	7	0.1	93.31%	90.83%
DRMA-DAML (ours)	7	0.5	94.62%	91.02%
DRMA-DAML (ours)	7	1.0	95.21%	92.34%
DRMA-DAML (ours)	7	2.0	93.21%	91.34%
DRMA-DAML (ours)	7	10.0	93.56%	91.27%
DRMA-DAML${\,}_{++F}$ (ours)	3	-	93.24%	90.89%
DRMA-DAML${\,}_{++C}$ (ours)	3	-	95.51%	92.26%
DRMA-DAML${\,}_{++F}$ (ours)	5	-	96.33%	94.12%
DRMA-DAML${\,}_{++C}$ (ours)	5	-	**97.64**%	**95.34**%
DRMA-DAML${\,}_{++F}$ (ours)	7	-	95.63%	93.94%
DRMA-DAML${\,}_{++C}$ (ours)	7	-	97.29%	93.51%

**Table 2:** Using human demonstrations as support tasks to train and test the model in Test Set 2. † denotes the ablation study, representing the model without deformable convolutions. N indicates a pick-and-place task on a non-complex background. C indicates a pick-and-place task on a complex background. ‘${\,}_{F}$’ and ‘${\,}_{C}$’ indicate the test results on the FCL head and CL head, respectively.

Method	Hyperparameters		N	C
	*n*	d		
DAML [15,19]	-	-	75.74%	70.42%
TaR-MIL [16,19]	-	-	68.89%	66.46%
MLMA-DAML [19]	3	0.5	78.64%	72.87%
MLMA-DAML [19]	5	0.5	81.37%	75.73%
MLMA-DAML${\,}_{++F}$ [19]	7	0.5	79.63%	74.12%
MLMA-DAML${\,}_{++C}$ [19]	7	0.5	83.54%	74.40%
DRMA-DAML${\,}_{F}^{†}$ [19]	5	1.0	86.25%	84.53%
DRMA-DAML${\,}_{C}^{†}$ [19]	5	1.0	87.23%	85.76%
DRMA-DAML${\,}_{C}$ (ours)	3	0.1	87.23%	83.35%
DRMA-DAML${\,}_{C}$ (ours)	3	0.5	88.92%	83.94%
DRMA-DAML${\,}_{C}$ (ours)	3	1.0	93.34%	90.44%
DRMA-DAML${\,}_{C}$ (ours)	3	2.0	91.37%	88.34%
DRMA-DAML${\,}_{C}$ (ours)	3	10.0	90.65%	85.49%
DRMA-DAML${\,}_{F}$ (ours)	5	0.1	90.91%	82.11%
DRMA-DAML${\,}_{C}$ (ours)	5	0.1	92.45%	86.51%
DRMA-DAML${\,}_{F}$ (ours)	5	0.5	91.88%	88.00%
DRMA-DAML${\,}_{C}$ (ours)	5	0.5	93.34%	89.55%
DRMA-DAML${\,}_{F}$ (ours)	5	1.0	92.69%	90.09%
DRMA-DAML${\,}_{C}$ (ours)	5	1.0	**94.87**%	**90.25**%
DRMA-DAML${\,}_{F}$ (ours)	5	2.0	91.74%	87.14%
DRMA-DAML${\,}_{C}$ (ours)	5	2.0	93.36%	89.19%
DRMA-DAML${\,}_{F}$ (ours)	5	10.0	92.46%	88.16%
DRMA-DAML${\,}_{C}$ (ours)	5	10.0	94.83%	89.90%
DRMA-DAML (ours)	7	0.1	91.38%	88.00%
DRMA-DAML (ours)	7	0.5	93.19%	89.06%
DRMA-DAML (ours)	7	1.0	93.84%	88.31%
DRMA-DAML(ours)	7	2.0	93.26%	89.85%
DRMA-DAML (ours)	7	10.0	94.41%	88.65%
DRMA-DAML${\,}_{++F}$ (ours)	3	-	90.76%	86.39%
DRMA-DAML${\,}_{++C}$ (ours)	3	-	92.45%	86.53%
DRMA-DAML${\,}_{++F}$ (ours)	5	-	93.82%	89.01%
DRMA-DAML${\,}_{++C}$ (ours)	5	-	**94.96**%	**90.34**%
DRMA-DAML${\,}_{++F}$ (ours)	7	-	92.24%	88.34%
DRMA-DAML${\,}_{++C}$ (ours)	7	-	94.53%	88.78%

**Table 3:** Using human demonstrations as support tasks to teach robots during training and robotic demonstrations as support tasks to teach robots during testing. ‘${\,}_{F}$’ and ‘${\,}_{C}$’ indicate the test results on the FCL head and CL head, respectively.

Method	Hyperparameters		Success Rate
	*n*	d	
DAML [15,19]	-	-	64.17%
TaR-MIL [16,19]	-	-	54.38%
MLMA-DAML [19]	3	0.5	68.34%
MLMA-DAML [19]	5	0.5	69.17%
MLMA-DAML${\,}_{++F}$ [19]	5	-	63.13%
MLMA-DAML${\,}_{++C}$ [19]	5	-	67.92%
MLMA-DAML${\,}_{++F}$ [19]	7	-	63.34%
MLMA-DAML${\,}_{++C}$ [19]	7	-	69.59%
DRMA-DAML${\,}_{F}$ (ours)	5	1.0	80.65%
DRMA-DAML${\,}_{C}$ (ours)	5	1.0	**82.33**%
DRMA-DAML${\,}_{++F}$ (ours)	5	-	82.32%
DRMA-DAML${\,}_{++C}$ (ours)	5	-	**82.89**%

**Table 4:** Using human and robotic demonstrations as support tasks to train the model, then testing it independently in Test Set 2 and Test Set 3. “Avg” represents the average success rate of learning from human and robotic demonstrations; α and β denote human and robotic demonstrations. ‘${\,}_{F}$’ and ‘${\,}_{C}$’ indicate the test results on the FCL head and CL head, respectively.

Methods	Random Factors		Test Set 2	Test Set 3	Avg
	α	β			
DAML [15,19]	1	1	65.28%	56.78%	61.03%
TaR-MIL [16,19]	1	1	68.21%	71.87%	70.04%
RDAML [17]	1	1	64.49%	64.68%	64.59%
RDAML [17]	1	[0, 0.3]	80.53%	55.88%	68.21%
RDAML [17]	1	[0, 0.5]	64.49%	64.13%	72.88%
RDAML [17]	[0, 1]	[0, 0.3]	74.81%	70.13%	72.47%
RDAML [17]	[0, 1]	[0, 0.5]	73.13%	77.09%	75.11%
RDAML [17]	[0, 1]	[0, 1]	73.15%	85.96%	79.55%
RDAML [17]	[0, 2]	[0, 2]	73.74%	78.84%	76.29%
DRMA-DAML${\,}_{++F}$	[0, 1]	[0, 1]	86.46%	98.97%	92.72%
DRMA-DAML${\,}_{++C}$	[0, 1]	[0, 1]	**90.32**%	**99.67**%	**95.00**%

**Table 5:** T-test results for MLMA-DAML vs. DRMA-DAML.

Test Set	MLMA-DAML Mean (%)	DRMA-DAML Mean (%)	*p*-Value
Test Set 1	83.92	90.32	0.014
Test Set 2	82.10	88.75	0.019
Test Set 3	79.56	85.94	0.031

## Data Availability

Our dataset can be found at https://github.com/CQUwilliam/Meta_learning_dataset (accessed on 5 December 2024).

## Abbreviations

The following abbreviations are used in this manuscript:

CL	Convolutional Layer
CNN	Convolutional Neural Network
D435	RealSense D435 Camera
DAML	Domain-Adaptive Meta-Learning
DCN	Deformable Convolutional Network
DRMA	Domain Residual Multi-Attention
DRMA-DAML	Domain Residual Multi-Attention Domain-Adaptive Meta-Learning
FC	Fully Connected
FCL	Fully Connected Layer
KAM	Kernel Attention Mechanism
LSTM	Long Short-Term Memory
MAML	Model-Agnostic Meta-Learning
MAML-DAML	Model-Agnostic Meta-Learning for Domain-Adaptive Meta-Learning
MIL	Meta-Imitation Learning
MLMA	Multi-Level Multi-Attention
MLMA-DAML	Multi-Level Multi-Attention Domain-Adaptive Meta-Learning
RDAML	Random Domain-Adaptive Meta-Learning
RTMAML	Replayed Task Contrastive Model-Agnostic Meta-Learning
SGD	Stochastic Gradient Descent
TaR-MIL	Target Recognition-Based Meta-Imitation Learning
